# Supplementary material for: Healthcare Practitioners Knowledge of Shoulder Injury Related to Vaccine Administration (SIRVA)
Source: Vaccines (Basel). 2022 Nov 23;10(12):1991. doi: 10.3390/vaccines10121991 (PMC9787830; doi:10.3390/vaccines10121991)
Supplement: Supplementary file 1 [file vaccines-10-01991-s001.zip › vaccines-2028547-supplementary.pdf]

Supplementary Materials

Qualtrics® questionnaire

Start of Block: Default Question Block

Q1 Participant Information Form
Project Title: Healthcare practitioner knowledge of Shoulder Injuries Related to Vaccine Administration (SIRVA).

Researcher
Name: Ms Laura Mackenzie
Faculty: Health
Email: u3198552@uni.canberra.edu.au

Supervisor
Name: Dr Mary Bushell
Phone: +61 2 62063934
Email: mary.bushell@canberra.edu.au

Project Aim:
The aim of this research is to investigate the current level of understanding held by healthcare professionals and healthcare students on the topic of Shoulder Injuries Related to Vaccine Administration (SIRVA), shoulder anatomy, and knowledge of immunisation safety and technique. MedDRA or Medical Dictionary for Regulatory Activities is an international medical terminology dictionary-thesaurus used by regulatory authorities. This study will also investigate if the current MedDRA definition of SIRVA is considered adequate by healthcare professionals.

Benefits of the Project:
The information gained from the research will be used to:
1. Determine current knowledge of SIRVA, its diagnosis, prevention, and treatment among healthcare professionals and healthcare students.
2. Determine the current level of shoulder anatomy knowledge relevant to vaccination precautions, among healthcare professionals and healthcare students.
3. Determine the current level of vaccination safety knowledge among healthcare professionals and healthcare students.
4. Gather information regarding vaccination techniques among healthcare professionals and healthcare students
5. Determine the preferred diagnostic criteria for SIRVA.

General Outline of the Project:
The project will consist of an online questionnaire using Qualtrics®, including a variety of question styles such as select all that apply, multiple choice, Likert scale, and heat map images.

Participant Involvement:
Participants who agree to participate in the research will be asked to:
1. Respond to questions on an online questionnaire
2. View photographs of individuals (male and female models) with limited clothing on the upper body

Participation in the research is completely voluntary and participants may, without any penalty, decline to take part or withdraw from the questionnaire at any time prior to completion.

Confidentiality:
Only the researcher/s will have access to the individual information provided by participants. Privacy and confidentiality will be assured at all times. The research outcomes may be presented at conferences and written up for publication. However, in all these publications, the privacy and confidentiality of individuals will be protected.

Anonymity:
All reports and publications of the research will not contain any information that can identify any individual,

and all information will be completely confidential.

Data Storage:
The information collected will be stored securely on a password protected computer throughout the project and then stored at the University of Canberra for the required five-year period after which it will be destroyed according to university protocols.

Ethics Committee Clearance:
The project has been approved by the Human Research Ethics Committee of the University of Canberra (HREC – 9192).

Queries and Concerns:
Queries or concerns regarding the research can be directed to the researcher and/or supervisor. Contact details are at the top of this form. If you have any complaints or reservations about the ethical conduct of this research, you may contact the University of Canberra's Research Ethics & Integrity Unit team via telephone 02 6206 3916 or email humanethicscommittee@canberra.edu.au or researchethicsandintegrity@canberra.edu.au
If you would like some guidance on the questions, or about your participation please refer to the Participants' Guide located at
http://www.canberra.edu.au/ucresearch/attachments/pdf/a-m/Agreeing-to-participate-in-research.pdf
OR email the student researcher Ms. Laura Mackenzie (u3198552@uni.canberra.edu.au) or primary supervisor Dr Mary Bushell (Mary.Bushell@canberra.edu.au)

Page Break

Q2 **Consent Form**
Healthcare practitioner knowledge of Shoulder Injuries Related to Vaccine Administration (SIRVA)
Consent Statement: I have read and understood the information about the research. I am not aware of any condition that would prevent my participation, and I agree to participate in this project. I have had the opportunity to ask questions about my participation in the research. All the questions I have regarding the research, have been answered to my satisfaction.
Future research may include but are not limited to: conferences, journal articles, discussions related to consensus definitions, or other publication formats.
Please indicate whether you agree to participate in each of the following parts of the research (please indicate which parts you agree to by putting a cross in the relevant box):

- ☐ Complete a 15 minute questionnaire (1)
- ☐ Agree to the anonymous information being used in future research (2)
- ☐ I do not agree to participate (3)

Skip To: End of Survey If Consent Form Healthcare practitioner knowledge of Shoulder Injuries Related to Vaccine Administr... = I do not agree to participate

---

Page Break

Q3 Where did you complete your training (for primary healthcare degree).

- Australia (1)
- Canada (2)
- New Zealand (3)
- United Kingdom (4)
- United States of America (5)
- Other (Please type answer below) (6) ____

Q4 Country of practice

- ○ Australia (1)
- ○ Canada (2)
- ○ New Zealand (3)
- ○ United Kingdom (4)
- ○ United States of America (5)
- ○ Other (Please type answer below) (6) ____

Q5 Field of practice

- ☐ Chinese Medicine (1)
- ☐ Chiropractic (2)
- ☐ Medicine (3)
- ☐ Medical Radiation Practice (4)
- ☐ Nursing & Midwifery (5)
- ☐ Osteopathy (6)
- ☐ Paramedicine (7)
- ☐ Pharmacy (8)
- ☐ Physiotherapy/Physical Therapy (9)
- ☐ Physicians assistant (10)
- ☐ Other (Please type answer below) (11) ____

Q6 I am:

- Fully qualified in my profession (1)
- A student working towards qualification (2)

Q7 I am an authorised vaccinator/immuniser

- Yes (1)
- No (2)

Skip To: Q11 If I am an authorised vaccinator/immuniser = No

Q8 Have you received vaccination training for intramuscular deltoid injections

- Yes, incorporated within an undergraduate degree (1)
- Yes, in a separate course to a degree (2)
- No (3)

Display This Question:

If Have you received vaccination training for intramuscular deltoid injections != No

Q9 Where did you complete your training (Vaccination specific)

- Australia (1)
- Canada (2)
- New Zealand (3)
- United Kingdom (4)
- United States of America (5)

Q10 Time spent in vaccination practice, in nearest whole year

____

Q11 I have heard the term Shoulder Injury Related to Vaccine Administration (SIRVA) before

- [ ] Yes (1)
- [ ] No (2)
- [ ] Yes, but by a different name (Please type answer below) (3)
____
- [ ] No, but possibly by a different name (Please type answer below) (4)
____

Skip To: Q13 If I have heard the term Shoulder Injury Related to Vaccine Administration (SIRVA) before = No

Q12 Where have you heard the term SIRVA (or an alternative term)

- [ ] Practice newsletter (1)
- [ ] Academic journal (2)
- [ ] University degree content (3)
- [ ] Other practitioners (4)
- [ ] Other (Please type answer below) (5) ____

Page Break

Q13 Click all that apply: What benefits are there to using landmarking techniques

- ▢ Provides an accurate site for injection (1)
- ▢ Protects the vaccinator from liability (2)
- ▢ Protects important underlying structures from being damaged (3)
- ▢ Improves patient trust in the practitioner (4)
- ▢ No benefits (5)
- ▢ Other benefits (Please type answer below) (6) ____

Q14 Click all that apply: What limitations are there to using landmarking techniques

- ▢ Time consuming (1)
- ▢ Inaccurate (2)
- ▢ Unnecessary, eyeballing is sufficient (3)
- ▢ Not effective at protecting underlying structures (4)
- ▢ No limitations (5)
- ▢ Other (Please type answer below) (6) ____

Page Break

Q15 The following questions refer to Shoulder Injury Related to Vaccine Administration (SIRVA), a very rare adverse event following vaccination. Standard vaccination involves injection into the deltoid muscle bulk, however SIRVA may occur when the vaccine is delivered into or close to surrounding structures of the upper arm.
Please answer the following questions to the best of your knowledge.

Q16 What do you understand as being contributing causes of SIRVA. Click all that apply.

- ☐ Incorrect needle length (1)
- ☐ Low body weight of patient (2)
- ☐ High body weight of patient (3)
- ☐ Incorrect administration site of vaccine (4)
- ☐ Vaccine ingredients (5)
- ☐ Local swelling resulting from vaccination (6)
- ☐ Genetics (7)
- ☐ Other (Please type answer in the text box) (8)

Q17 Which structures surrounding the deltoid muscle do you understand could be damaged in SIRVA.

	Yes (15)	No (16)	Unsure (17)
Radial nerve (1)			
Axillary nerve (2)			
Ulnar nerve (3)			
Subacromial bursa (4)			
Subdeltoid bursa (5)			
Humerus (7)			
Acromion process (8)			
Rotator cuff tendons (18)			
Coracoacromial ligament (9)			
Shoulder capsule (10)			
Deltoid muscle (11)			
Radial artery (14)			
Brachial artery (16)			
Other (Please type answer below) (17)			

Q18 Which conditions do you understand may result from incorrect intramuscular injection technique.

	Yes (15)	No (16)	Unsure (17)
Bursitis (Inflammation of the bursa) (1)			
Osteonecrosis (Bone death) (2)			
Tendinopathy/Tendonitis (3)			
Arteriosclerosis (Narrowing of an artery) (4)			
Frozen shoulder (Adhesive capsulitis) (5)			
Shoulder impingement syndrome (6)			
Nerve palsy/paralysis (7)			
Nerve entrapment (8)			
Neuritis (Nerve inflammation) (9)			
Rotator cuff syndrome (10)			
Osteoarthritis (11)			
Synovitis (Capsular inflammation) (12)			
Fractures (13)			
Osteoporosis (14)			
Rheumatoid arthritis (15)			
Shoulder instability (16)			
Other (Please type answer below) (17)			

Display This Question:

If I am an authorised vaccinator/immuniser = Yes

Q19 For the questions below please mark how strongly you agree or disagree with each statement.

	Strongly disagree (1)	Disagree (2)	Somewhat disagree (3)	Somewhat agree (4)	Agree (5)	Strongly agree (6)	Not applicable (7)
I use landmarking techniques every time I inject into the shoulder (4)							
I have had landmarking techniques used on me, while receiving a shoulder injection (5)							
I feel vaccinators have adequate training regarding SIRVA (6)							
I feel confident in my understanding of shoulder anatomy (1)							
I feel confident in my understanding of safe injecting techniques (2)							
I feel confident in my training in safe injecting techniques (3)							
I feel confident in my ability to recognise SIRVA (7)							
I am confident referring a case of SIRVA to the appropriate healthcare professional for treatment (8)							
I am confident reporting a suspected case of SIRVA to the relevant governing body (9)							

Display This Question:

If I am an authorised vaccinator/immuniser = No

Q20 For the questions below please mark how strongly you agree or disagree with each statement.

	Strongly disagree (1)	Disagree (2)	Somewhat disagree [illegible]	Somewhat agree (4)	Agree (5)	Strongly agree (6)	Not applicable (7)
I feel healthcare professionals have adequate training regarding SIRVA (3)							
I feel confident in my understanding of shoulder anatomy (1)							
I feel confident in my understanding of safe injecting techniques (2)							
I feel confident in my ability to recognise SIRVA (4)							
I am confident referring a case of SIRVA to the appropriate healthcare professional for treatment (5)							
I am confident reporting a suspected case of SIRVA to the relevant governing body (6)							

Page Break

Q21 The following questions will present images of individuals upper arms, in various positions. For each question select (click or tap) the approximation of the area or structure requested to the best of your knowledge/ability, without the use of external sources.

Q22 Select the most appropriate area for intramuscular injection during vaccination.

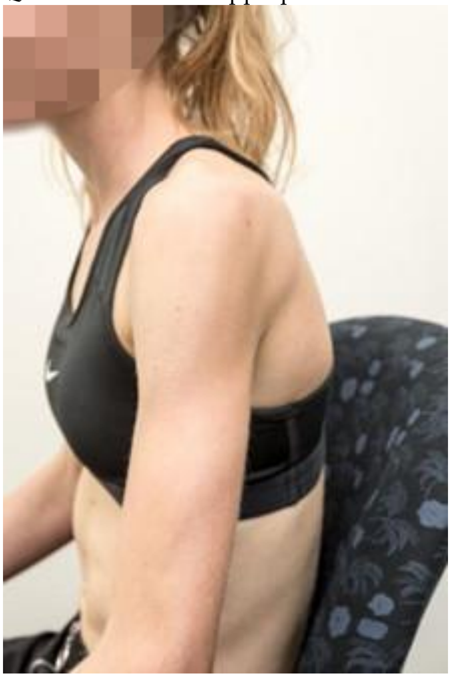

Q23 Click the approximate position of the acromion process.

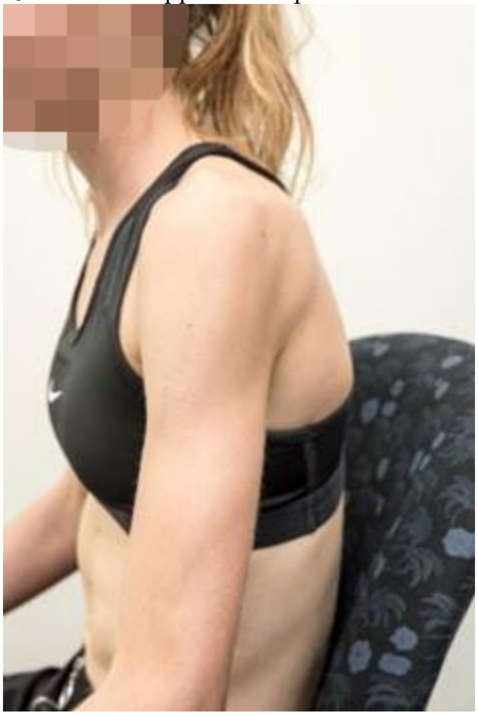

Q24 Click the approximate position of the axillary nerve.

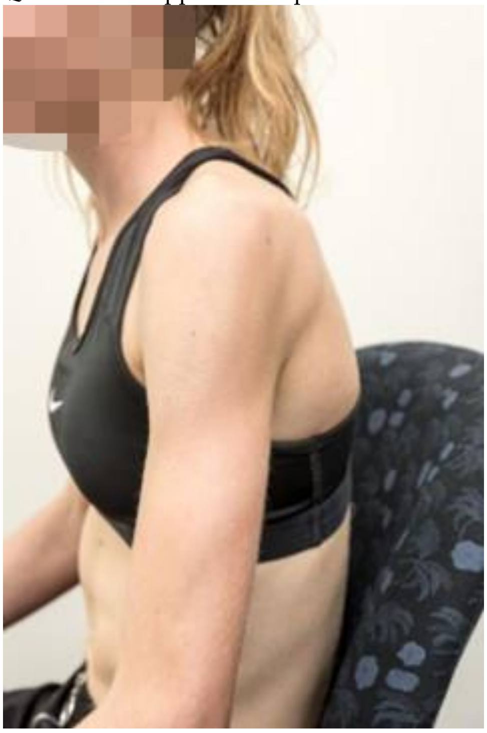

Q25 Click the approximate position of the radial nerve (where it is most superficial along the upper arm).

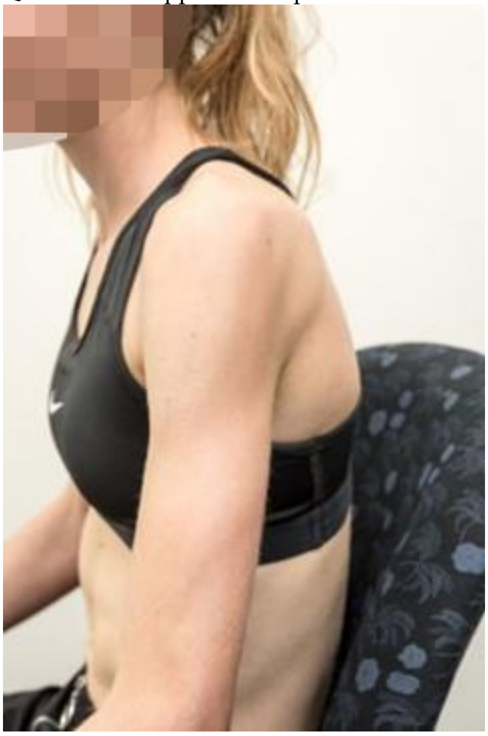

Q26 Click the approximate position of the deltoid tuberosity.

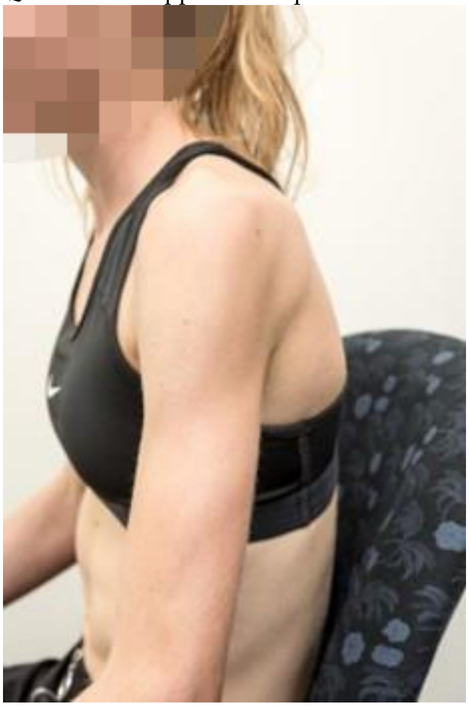

Q27 Click the approximate position of the subacromial/subdeltoid bursa.

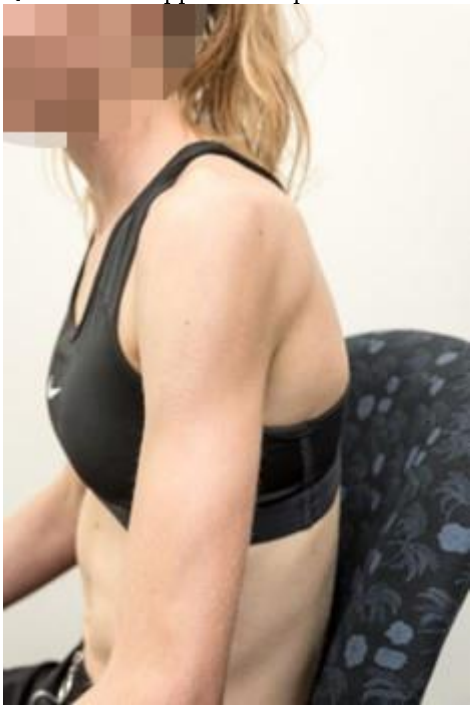

Page Break

Q28 The following questions will present images of either correct or incorrect examples of landmarking/immunisation techniques. Please nominate whether you identify them to be correct or incorrect.

Q29 Image 1

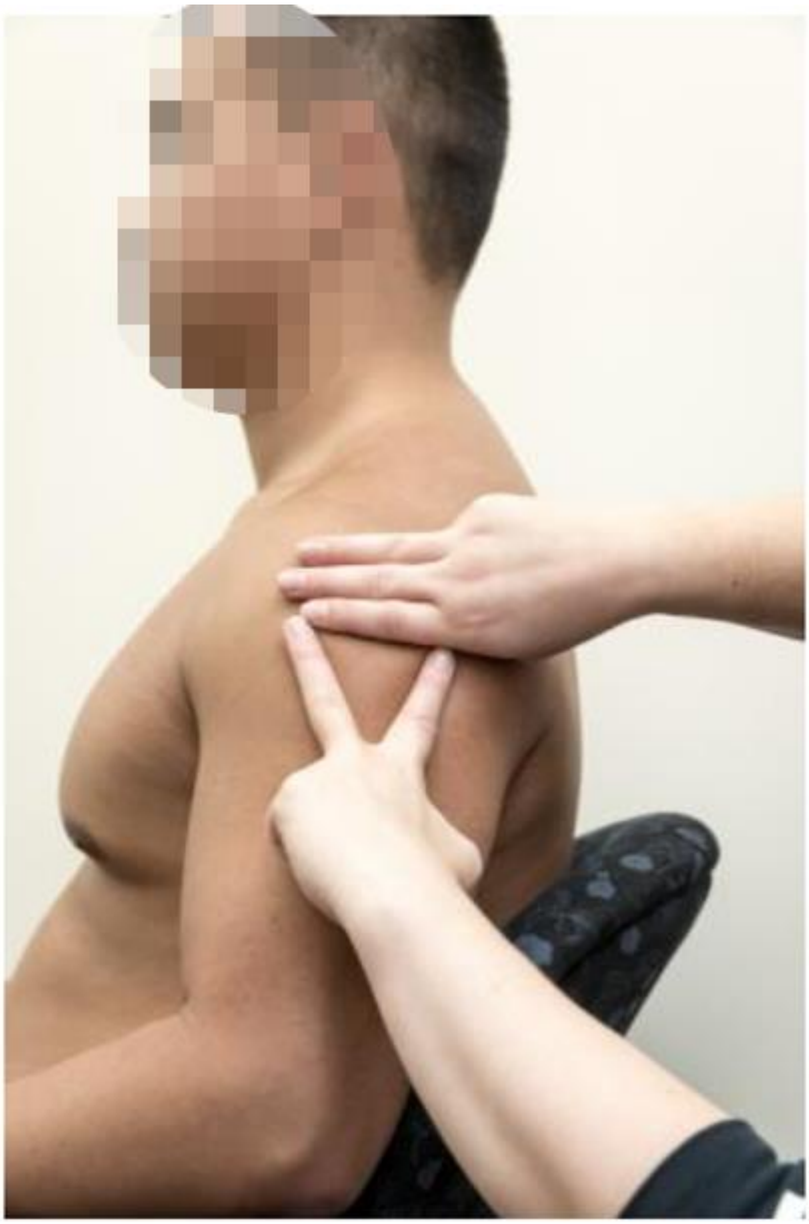

Q30 Image 1 is an example of _____ technique

○ Correct (1)

○ Incorrect (2)

Q31 Image 2

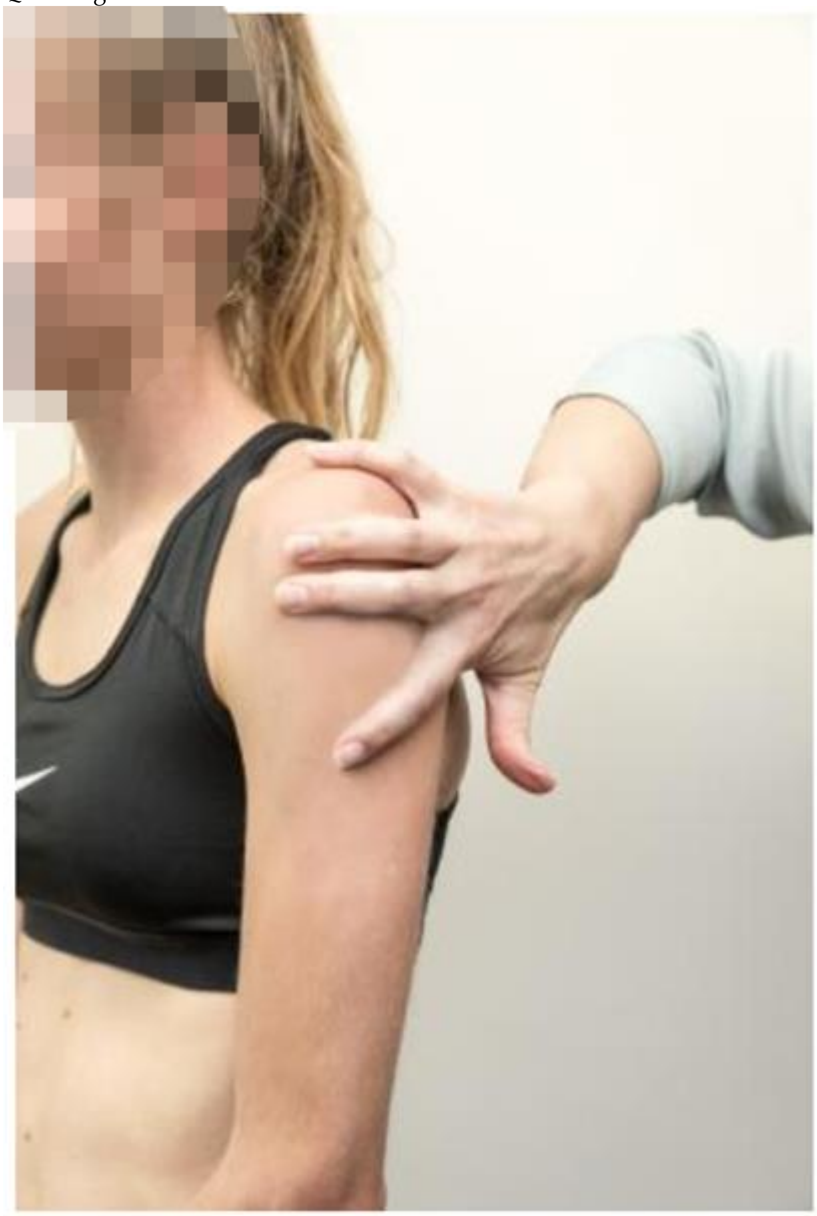

Q32 Image 2 is an example of _____ technique

- Correct (1)
- Incorrect (2)

Q33 Image 3

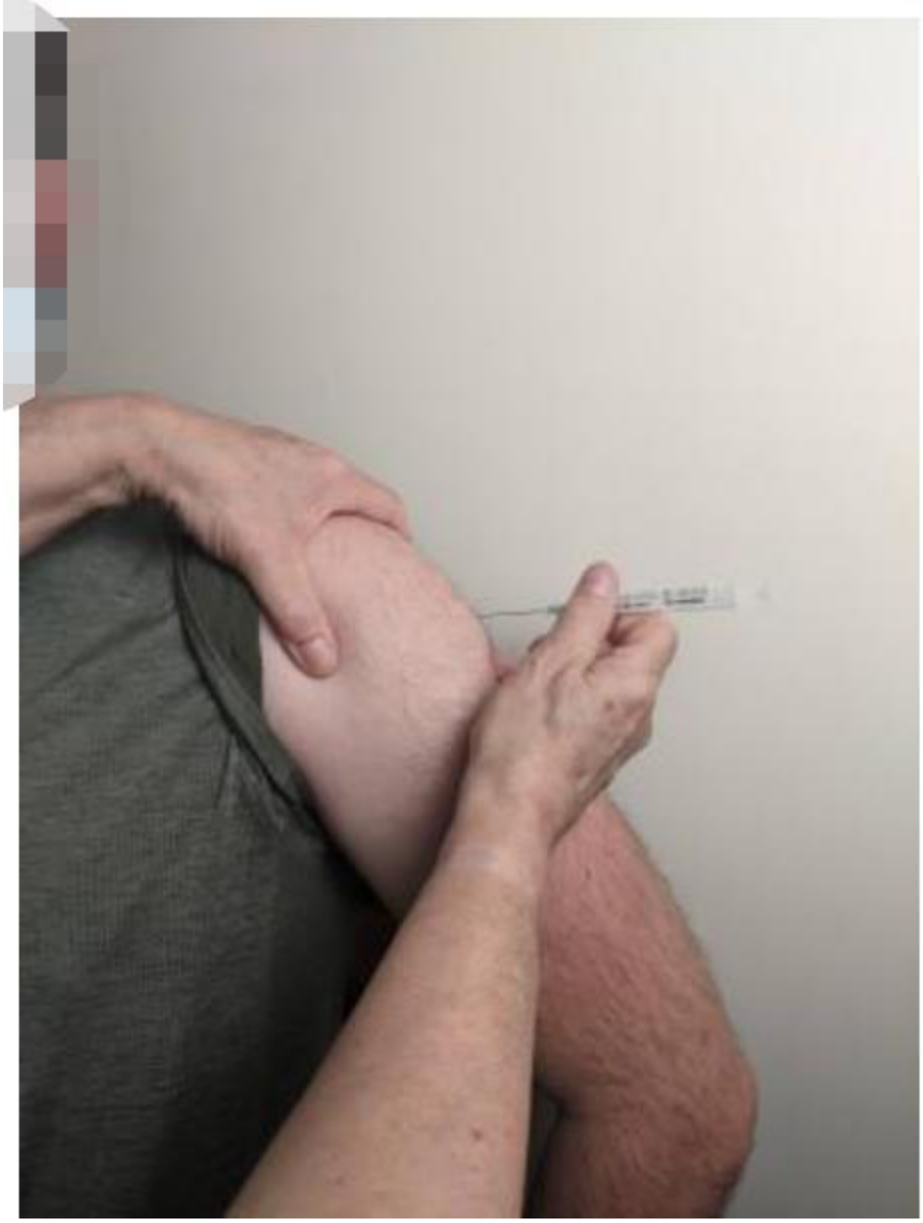

Q34 Image 3 is an example of _____ technique

- ◯ Correct (1)
- ◯ Incorrect (2)

Q35 Image 4

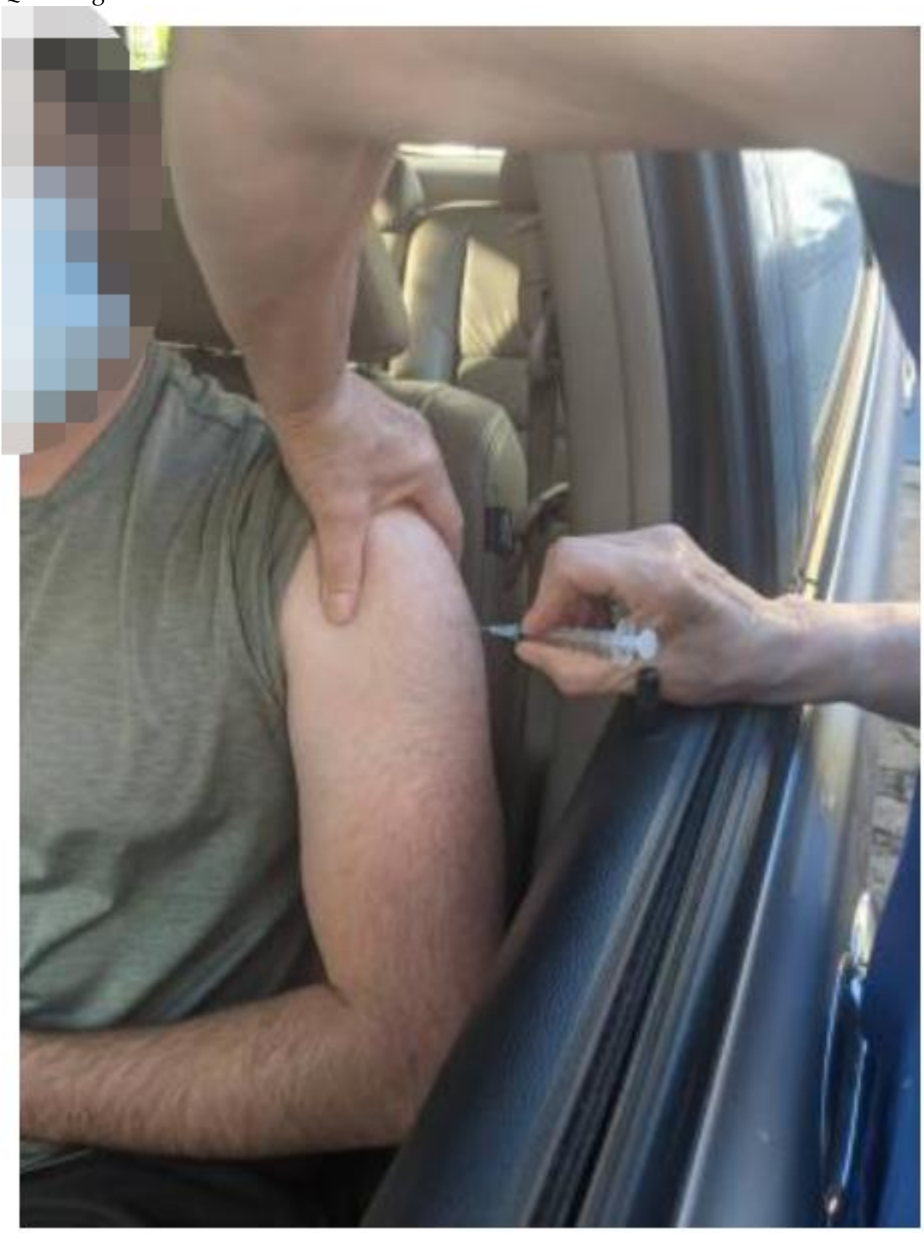

Q36 Image 4 is an example of _____ technique

○ Correct (1)

○ Incorrect (2)

Q37 Image 5

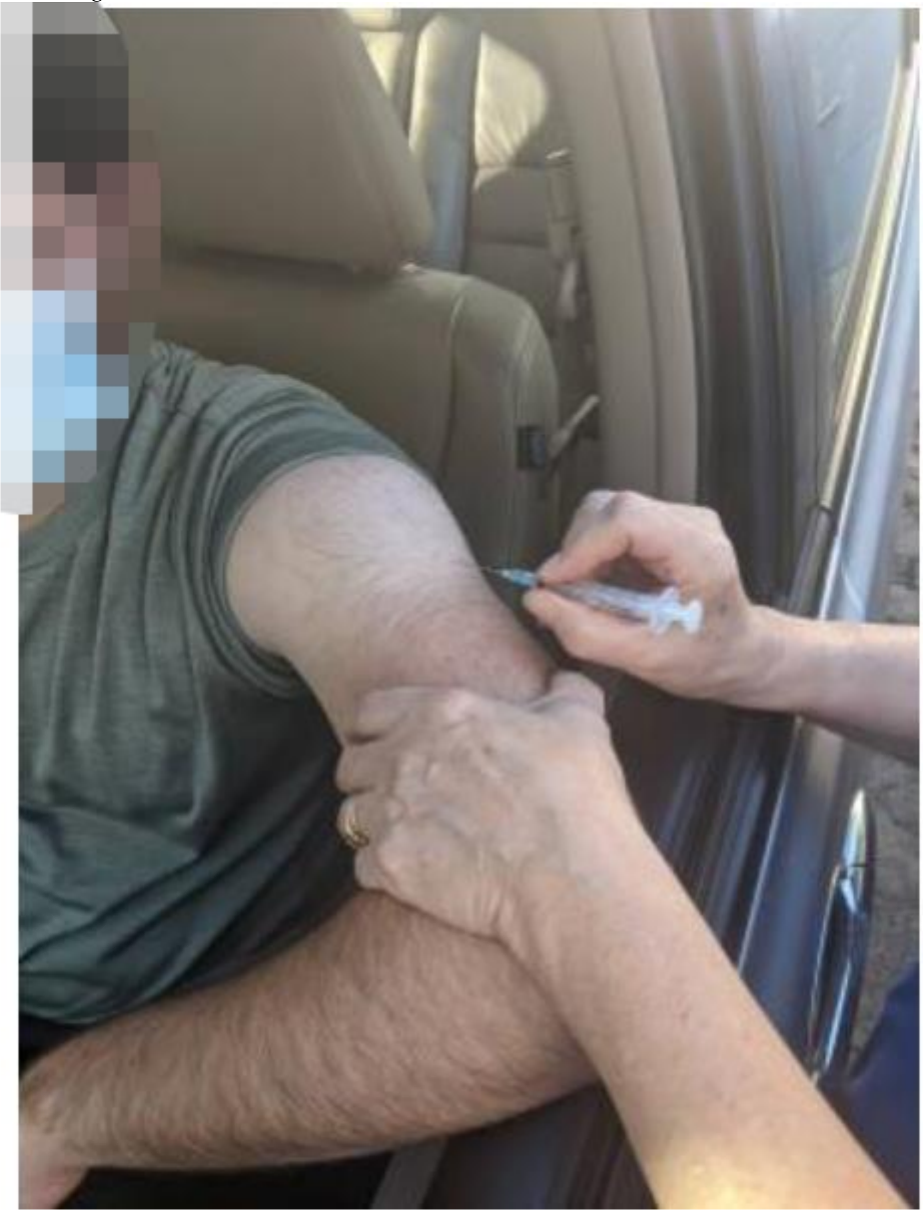

Q38 Image 5 is an example of _____ technique

- ○ Correct (1)
- ○ Incorrect (2)

Q39 Image 6

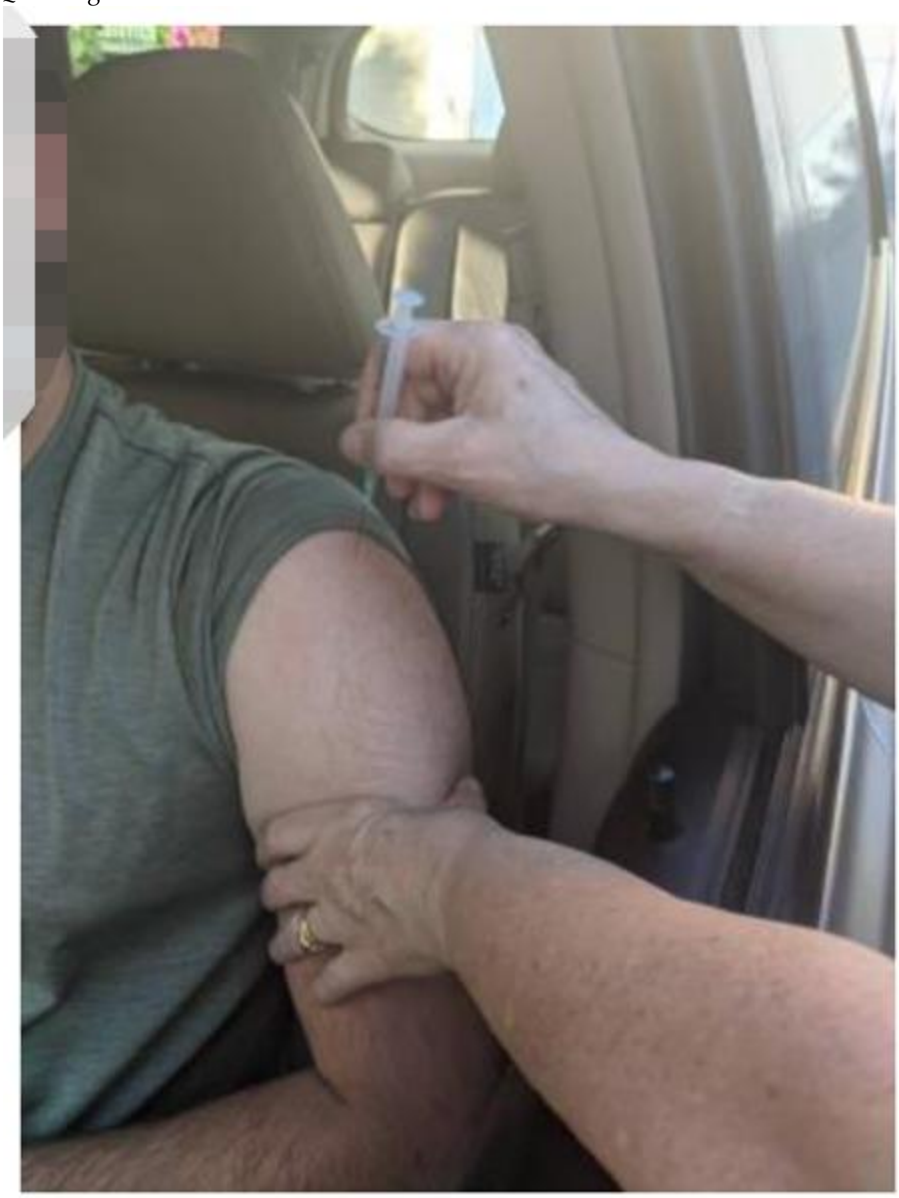

Q40 Image 6 is an example of _____ technique

○ Correct (1)

○ Incorrect (2)

Q41 Image 7

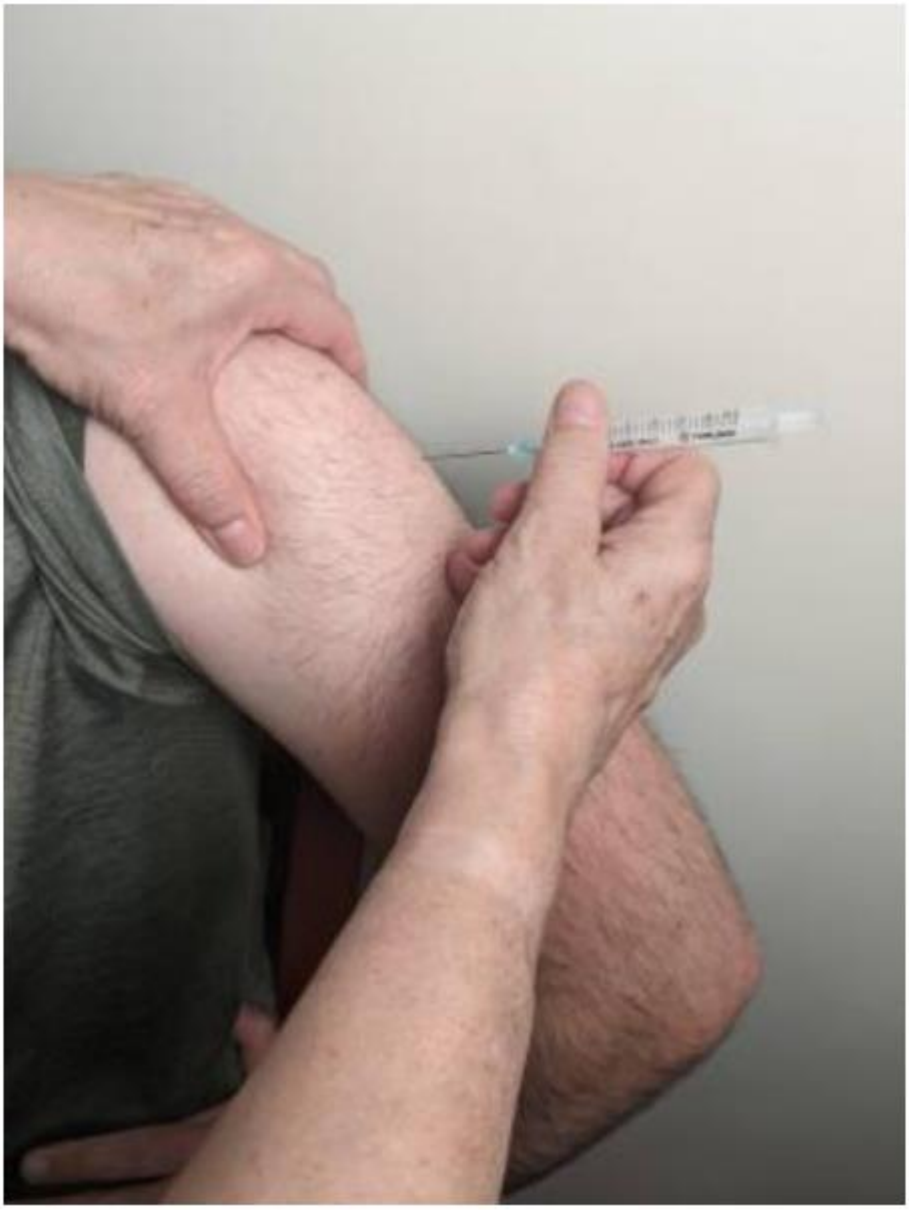

Q42 Image 7 is an example of _____ technique

- Correct (1)
- Incorrect (2)

Q43 Image 8

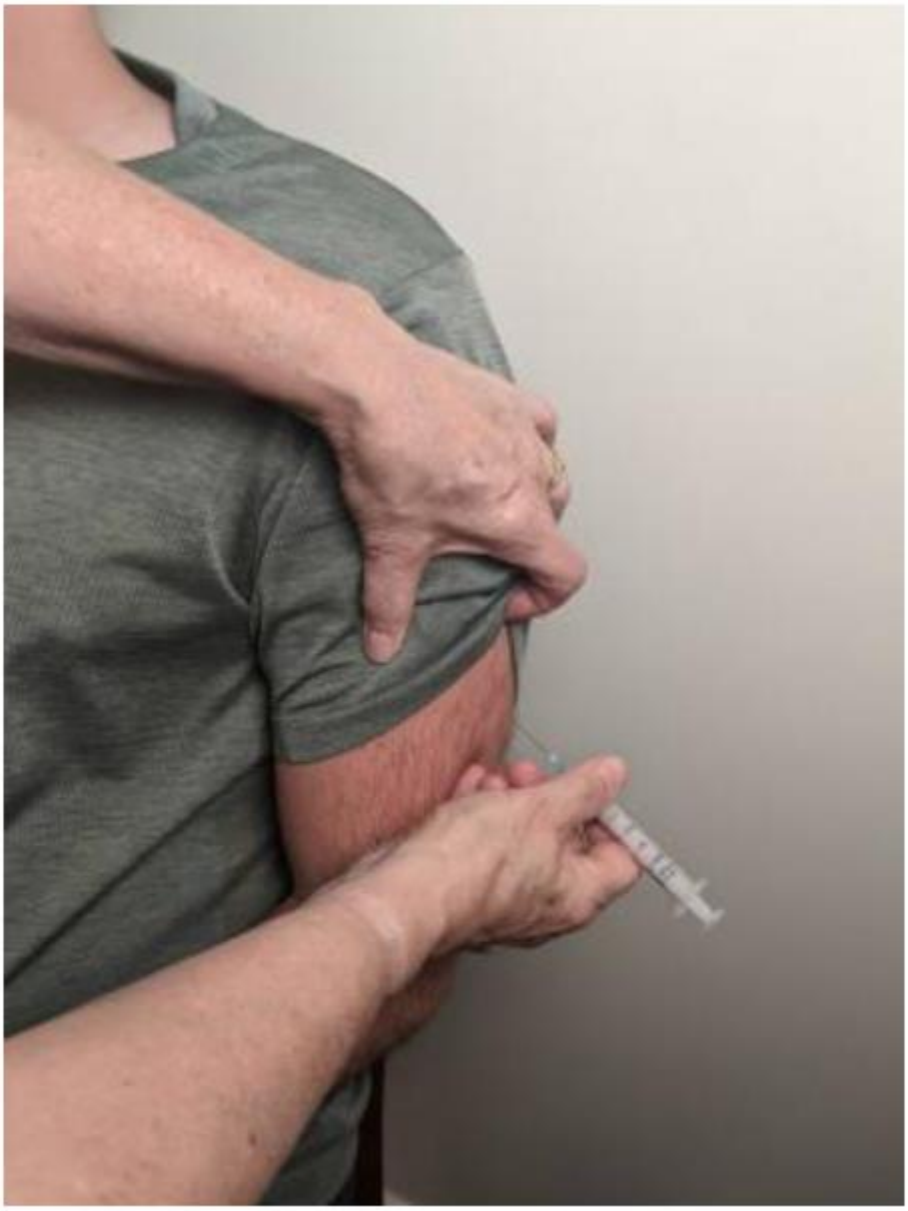

Q44 Image 8 is an example of _____ technique

○ Correct (1)

○ Incorrect (2)

Q45 Image 9

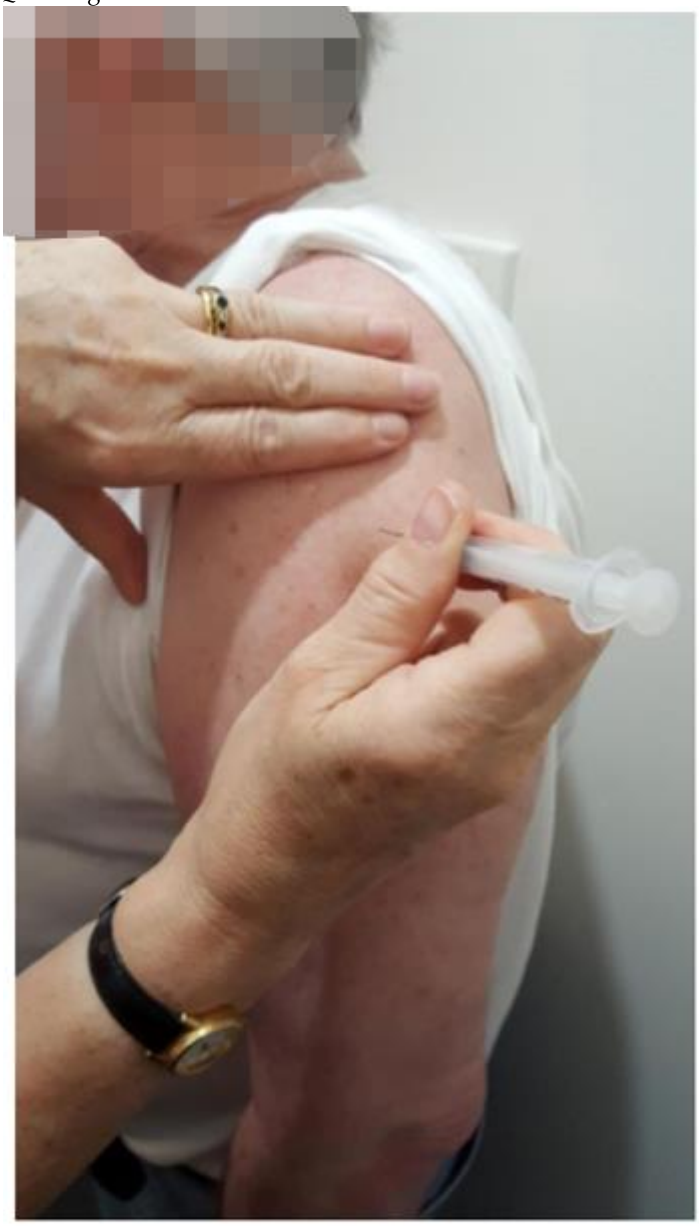

Q46 Image 9 is an example of _____ technique

- ○ Correct (1)
- ○ Incorrect (2)

Page Break

Q47 The following questions relate to the two most commonly used definitions of SIRVA.

Q48
SIRVA definition A:
1. Pain occurs within 48 hours
2. Pain and reduced range of motion/movement, limited to the limb of the injection site
3. No prior history of pain, inflammation, or dysfunction
4. No neurological injuries or abnormalities
5. No other differential diagnoses present

SIRVA definition B:
1. Onset of symptoms within 24-48 hours
2. Pain on movement
3. Restricted range of motion of affected limb
4. Abnormalities present on medical imaging
5. Suspicion of incorrect technique of vaccine administration

Using the above definitions, select which you feel most appropriately defines SIRVA.

○ A (1)

○ B (2)

○ Both A and B are appropriate (3)

○ Neither are appropriate (please indicate reasons below) (4)
____

Q49 Definition A excludes neurological injuries (nerve injuries) from being SIRVA. Do you think nerve injuries to the upper arm following injections, should be considered as SIRVA?

○ Yes (1)

○ No (2)

Q50 Participants may enter an email below to have the chance to win one of two $50 AUD gift cards (or nearest equivalent amount based on exchange rates to desired currency). Gift cards will be distributed via email. Participants may also opt to receive information regarding the outcome of the study on completion.
I would like to:

▢ Go in the chance to win a $50 AUD or equivalent gift card (1)

▢ Receive information regarding the outcome of the study on completion (2)

▢ Decline receiving information and going in the gift card draw (3)

Display This Question:

If Participants may enter an email below to have the chance to win one of two $50 AUD gift cards (or... != Decline recieving information and going in the gift card draw

Q51 Please enter a valid email below:

○ Email (1) ____

End of Block: Default Question Block
